# “Now that I took TPT, it’s affecting my ART adherence, viral load, even my wellbeing in the community”. Exploring acceptability and experience of Tuberculosis Preventive Treatment among adolescents living with HIV in Zimbabwe

**DOI:** 10.1371/journal.pgph.0005102

**Published:** 2025-11-04

**Authors:** Joni Lariat, Rufaro Mbundure, Webster Mavhu, Abigail Mutsinze, Mikaela Coleman, Sharon Sibanda, Pueshpa Shaba, Laura Kafata, Leviticus Makoni, Ann Selberg, Carol Wogrin, Owen Mugurungi, Charles Sandy, Nicola Willis, Sarah Bernays

**Affiliations:** 1 Sydney School of Public Health, University of Sydney, Camperdown, New South Wales, Australia; 2 Centre for Sexual Health and HIV/AIDS Research (CeSHHAR), Harare, Zimbabwe; 3 Department of International Public Health, Liverpool School of Tropical Medicine, Liverpool, United Kingdom; 4 Zvandiri, Harare, Zimbabwe; 5 Ministry of Health and Childcare, Harare, Zimbabwe; 6 Africa Union Development Agency–NEPAD, Johannesburg, South Africa; University of California San Francisco, UNITED STATES OF AMERICA

## Abstract

Tuberculosis preventive treatment (TPT) is increasingly offered to people living with HIV in high-burden settings, including adolescents and young people (AYPLHIV). Evidence demonstrates that AYPLHIVs’ HIV treatment engagement is improved by the provision of tailored support. How to effectively adapt this support to accommodate multimorbidity care, such as TPT alongside ART, warrants attention to deliver sustained optimal outcomes. We conducted qualitative research to better understand AYPLHIVs’ experiences when initiating TPT and their related support needs. Peer counsellors (18–24 years) who were offered TPT within routine HIV care participated in two focus groups (n = 16 participants) and in-depth interviews (n = 12) in Harare. Iterative data collection and thematic analysis was conducted September 2023 to February 2024. TPT was presented by healthcare workers as uncomplicated and routine. This contrasted with participants’ accounts of the significant disruptive and challenging experience of taking up TPT. This tension led many to stop TPT without support. Those who completed treatment were motivated by personal circumstances (e.g., recently witnessing severe TB illness, pregnancy); however, taking up and completing subsequent courses of TPT was not assured. TPT side effects and stigma led many to discontinue treatment, even when these were not personally experienced. Side effects recalled past experiences of HIV-stigma and discrimination, and undermined ART adherence, HIV viral suppression, and positive mental health. Introducing additional life-protecting treatments can have complicating biosocial effects, with consequences for individuals and public health. To support multimorbidity prevention and care, we outline five principles to guide initiating and maintaining treatment that acknowledges and responds to AYPLHIVs’ dynamic immunological and social realities: Supported and safe relationships; Tailored messaging; Adaptable support; Respect for agency and autonomy; Timing: plan, review, revise (START). START emphasises investing in consistent peer support throughout adolescence, and centring AYPLHIVs’ agency, embodied knowledge and wellbeing to ensure they are informed decision-makers about their health.

## Introduction

Tackling HIV-TB comorbidity is critical for redressing health and economic inequalities globally [1]. The clinical progress in antiretroviral therapy (ART), particularly the more tolerable regimen, dolutegravir, has significantly improved HIV outcomes, including the number of people who are able to sustain viral suppression [2,3]. This progress has allowed greater attention to be directed towards the need for addressing multimorbidity by integrating differentiated services, including testing, treatment and prevention of TB, within HIV care [4–6].

In high burden settings, people who are living with HIV (PLHIV), including adolescents and young people (AYPLHIV; 10–24 years), are at heightened risk of HIV-TB coinfection and associated poor outcomes [7,8]. Despite progress in HIV management, evidence shows that AYPLHIV remain at high risk of developing TB, even when they are virally suppressed due to sustained ART adherence [7]. AYPLHIVs’ increased risk of progression from TB infection to disease, combined with the known poor outcomes of HIV-TB co-infection on mental and physical health, highlight the need for continued attention towards improving person-centred integrated care for AYPLHIV [9].

The challenges facing AYPLHIV are well-documented [10–13]. In addition to the broader diagnostic and treatment challenges associated with managing HIV-TB coinfection [14], for AYPLHIV, existing difficulties are amplified by the introduction of treatment for TB, which requires lengthy regimens that carry potential drug interactions [7]. It is imperative then that the TB treatment and prevention strategies that are integrated within HIV care are tailored to meet AYPLHIVs’ specific clinical and psychosocial needs.

Tuberculosis Preventive Treatment (TPT) significantly reduces mortality, morbidity, and incidence of TB among PLHIV [14–16]. Despite evidence of its cost-effectiveness and impact on key indicators, the implementation and scale-up of TPT in resource-limited settings has been slow [15,17,18], and while it is increasingly available to PLHIV in high-burden settings, uptake remains low [5,19,20]. As TPT is further integrated into routine HIV care, there is an opportunity to better understand how it interacts with HIV management for all PLHIV, including AYPLHIV.

Zimbabwe has a high burden of TB and TB/HIV coinfection, and TB remains the leading cause of death among PLHIV [21]. Despite the country’s transition out of the 30 high TB burden countries in 2021, it remains on the Global TB watchlist 2024 of countries requiring continued attention [21]. At the time of this study, Zimbabwe’s Ministry of Health and Child Care (MoHCC) adhered to the current WHO guidelines recommending TPT every three years for all PLHIV [22]. In response to growing consensus that there is no significant benefit in re-administering TPT, the MoHCC amended their guidelines in July 2024 (after this study had been completed) to recommend that PLHIV will be administered TPT only once, unless they become a contact of a bacteriologically confirmed TB case.

These changes are welcome. They will reduce the need for PLHIV to take TPT intermittently for the rest of their lives and, if accompanied by increased active case finding and testing, are unlikely to affect Zimbabwe’s progress in TB prevention and control. Given the challenges of managing frequent, simultaneous ART and TPT, these changes are likely to have the greatest benefit for AYPLHIV. Given AYPLHIV will still be offered TPT if they are exposed to TB, which is critical for TB prevention, it is imperative that we better understand their experiences and perceptions of TPT within the context of HIV management.

Recent studies in South Africa and Uganda have explored TPT regimen and service delivery preferences with both children and providers [23,24], and have identified key barriers and facilitators to TPT uptake and completion among children and adolescents who are living with HIV [25]. There are opportunities to further understand how TPT interacts with AYPLHIVs’ existing concerns and priorities whilst they manage HIV, and how peer support can be harnessed to ensure ART engagement and mental health are not compromised by engaging with additional treatments such as TPT.

In Zimbabwe, the rollout of TPT to AYPLHIV has been facilitated by their existing relationship and engagement with a leading peer-led differentiated support programme called Zvandiri (As I Am), which is operating at scale nationally. Zvandiri has been shown to significantly improve ART adherence, viral suppression, and mental health and wellbeing among children, adolescents and young people [26–28]. Psychosocial support is delivered by Zvandiri peer counsellors, titled community adolescent treatment supporters (CATS), who are themselves living with HIV [26,28,29]. While TPT is administered routinely as part of adolescents’ HIV care within clinical settings, Zvandiri’s peer support networks are being leveraged to provide psychosocial support around the delivery of integrated care and support for co-morbidities, such as TB.

Indicative of the effectiveness of the Zvandiri approach, a high proportion of AYPLHIV who are receiving Zvandiri peer support are virally suppressed [26]. However, evidence from a clinical trial suggests that the addition of TPT for people on ART can affect the consistency of ART use and/or viral suppression [30]. In line with Zvandiri’s commitment to providing differentiated support to AYPLHIV in navigating challenges affecting their ability to live well with HIV, there has been a growing interest in better understanding the challenges associated with TPT in the context of adolescent HIV management. This is part of a broader desire to learn how to support AYPLHIV to engage in health-enhancing and protective interventions for a range of potential co-morbidities without compromising HIV care stability.

Our longstanding collaborative partnership has allowed us to draw on in-depth understandings of the contexts that shape AYPLHIVs’ relationship with HIV, health, and wellbeing in a supportive and destigmatising environment. This provides a valuable foundation for examining the negotiation of TPT, an intervention aimed towards further protecting and enhancing health and wellbeing. Examining AYPLHIV’s perceptions and experiences of TPT offered an opportunity to explore the implementation and impact of integrated person-centred care under real-world conditions, beyond the limitations of a trial setting.

We conducted qualitative research with Zvandiri peer counsellors to learn about their experiences of TPT and providing support to those in their care who were also navigating TPT. The study aimed to develop more nuanced understandings of how TPT is prioritised by AYPLHIV alongside their broader life concerns and health negotiations. It was intended that these insights would principally inform future Zvandiri programming to provide differentiated support for AYPLHIV who are offered TPT. It also aimed to raise important considerations for how to approach conversations about additional health-promoting treatments with all people who are living with HIV and who may have fragile or unstable ART adherence. These intersecting areas of inquiry are vitally important as preventive treatments for a range of co-morbidities become increasingly available.

## Methods

### Study setting

This qualitative study was carried out in Harare, Zimbabwe, where TPT was first introduced in 2012 and expanded by 2018 for PLHIV. Until 2020, a six-month daily isoniazid (INH) regimen [31,32] was administered alongside pyridoxine to prevent neuropathy, one of INH’s most common major side effects. Other side effects include skin blemishes (e.g., rash), gastrointestinal issues, weakness, and hepatotoxicity (i.e., liver damage). In 2020, the 3HP regimen (a once-weekly dose of rifapentine and isoniazid taken for three months) was introduced, with TPT scale-up taking place between 2022–2023. The shorter duration and lower frequency of 3HP have largely improved tolerability and acceptability, despite its common side effects. While it carries a lower risk of hepatotoxicity compared to daily isoniazid, 3HP is associated with a higher frequency of systemic drug reactions, such as fatigue, dizziness and flu-like symptoms. For PLHIV, the combination of ART and rifapentine can lead to such drug reactions [33].

Until July 2024, TPT was readministered every three years as part of routine HIV care, regardless of the regimen. By 2024, guidelines were revised to recommend prioritising TPT for individuals who had not previously received it, with re-administration reserved for those with known exposure to a confirmed TB case. All data collection for this study was completed before July 2024. All participants were offered TPT as part of routine care, and expected that this would continue every three years for the rest of their lives.

### Study participants, sampling and recruitment

Study participants were Zvandiri peer counsellors (CATS; 18–24 years) who are living with HIV. All participants had been offered TPT at least once during adolescence and had been CATS for at least one year. In their role as CATS, they had all received TB-specific training, and routinely provided TPT guidance to Zvandiri beneficiaries. Participants were purposively sampled to ensure a representative sample of Zvandiri CATS in terms of age, gender and varied known experiences with TPT, including those who had completed or discontinued treatment for a range of reasons. Programme data were used to assist Zvandiri staff in identifying and recruiting potential participants. Once identified, the research team contacted each participant by phone to explain the research focus and aims and to invite their participation in the study. All CATS who were contacted agreed to participate.

### Data collection

We conducted iterative data collection and analysis between September 2023 and February 2024. Mixed qualitative methods (focus group discussions (FGDs) and individual in-depth interviews (IDIs)) were used to explore the social dimensions of TPT and how it is perceived and experienced by AYPLHIV. We began with FGDs to explore how TPT was socially constructed and talked about by AYPLHIV in the context of both their own uptake and their roles as peer supporters. As a research team, we have been engaging Zvandiri beneficiaries and peer counsellors on topics related to HIV for many years, providing us with a deep understanding and familiarity with how this group typically discusses HIV-related challenges and experiences. The FGDs allowed us to observe whether there were any different sensitivities in speaking openly about TPT and the challenges it presents to AYPLHIV. We held two FGDs, each with participants with known similarities in experience, to promote the safe sharing of experiences and decision-making related to TPT. FGD 1 included participants who had taken up TPT and were continuing or had concluded treatment (n = 7). FGD 2 included participants who had been offered TPT but had not begun treatment or had discontinued prior to completion (n = 9). This design also facilitated comparative analysis of key themes related to TPT acceptability between the two groups, which we presumed had contrasting TPT journeys and outcomes. The FGDs lasted approximately 1.5 hours and followed a semi-structured guide developed for each group based on qualitative literature and our contextual understandings (S1 and S2 Text).

We then conducted IDIs to gain deeper insight into participants’ individual experiences and to ensure that perspectives that may not have been easily discussed during the FGDs could be safely shared. 9/16 (56%) FGD participants expressed an interest in participating in an IDI (4/7 from FGD 1, and 5/9 from FGD 2). One participant who could not attend an FGD was interviewed, as well as two further participants who were purposively selected to enrich our understanding of emergent topics. The same inclusion criteria and recruitment process were applied. Iterative analysis throughout data collection informed ongoing refinement of the interview topic guide (S3 Text). Data collection was concluded once we were satisfied with our breadth and depth of understanding.

FGDs and IDIs were conducted in Shona (the participants’ language) by a trained qualitative researcher (RM). Interviews were audio-recorded, transcribed and translated into English, and transcripts were checked by a second researcher for consistency. RM created fieldnotes immediately after each data collection event to capture immediate impressions and contextual information.

### Data analysis

Reflexive thematic analysis was conducted throughout data collection. Given our interest in better understanding both the lived experiences and social meanings of TPT, thematic analysis was an appropriate analytical technique for comparing and analysing data collected from FGDs and IDIs. Divergences in participants’ accounts across these two methods were explored for what this could tell us about how TPT is socially constructed and experienced.

The analytical process involved two analysts (JL and RM) independently familiarising themselves with the data as it was transcribed. Coding and memo writing identified and explored key topics and areas of significance that could be further discussed during weekly analytically focused meetings with other members of the research team (SB, WM, PS, SS). These meetings were a vital component of the analytical process, providing a space to collectively review and robustly discuss initial codes, and to reflect critically on how our positionalities shaped our interpretations. Specific attention was paid to outlier cases to explore the contextual and circumstantial specificity that shaped participants’ experiences and perspectives. This process of independent analysis and collective discussion throughout data collection facilitated a growing consensus on how codes could be collated, condensed, and eventually elevated to themes and sub-themes. Once these were defined and illustrated with exemplar quotes, the wider authorship team was invited to share their perspectives and expertise on the programmatic implications of the findings.

### Ethics statement

Ethics approval was provided by the Medical Research Council of Zimbabwe (#A/2860). Participants provided written informed consent to participate in an audio-recorded focus group discussion and/or individual interview. The study aims and confidentiality procedures, as well as the potential risks and benefits, were discussed with participants at the time of consenting. Participants’ quotations have been deidentified and are labelled using a participant identification (PID) number. Each participant received US$10 bus fare reimbursement.

### Inclusivity in global research

Additional information regarding the ethical, cultural and scientific considerations specific to inclusivity in global research is provided in the S1 Checklist.

## Results

All 19 Zvandiri peer counsellors who were invited agreed to participate in the study (Table 1). Of the participants, 11 identified as women and eight as men, with ages ranging from 19 to 24 years (mean age: 22.2). All participants had been working as peer counsellors for at least one year, with a large majority (89%) having served in the role for over two years. All had completed peer counsellor training, which included specific guidance related to TPT. Of those interviewed (n = 12) five (42%) said they had completed their most recent course of TPT. For the seven who had not completed their most recent course of TPT, five (71%) cited side effects as the major reason for discontinuing treatment.

**Table 1:** Participant characteristics.

Characteristic	N (%)
Gender, N (%) female	11 (57.9%)
Age, N (%) >20 years	18 (94.7%)
Duration as peer counsellors, N (%) >2 years	17 (89.4%)

The two FGDs, organised according to participants’ perceived completion of TPT, generated immediate insights. These discussions contrasted with the open conversations that this group typically has about HIV and their challenges with ART adherence. Participants appeared reluctant to share their experiences with TPT, especially if they had chosen to forgo or discontinue TPT. IDIs offered deeper insight into participants’ individual experiences with TPT, with many describing uncertainty about how to discuss TPT-related challenges with their peers.

IDIs also demonstrated that our delineation of the two FGD groups based on perceived completion, and, by inference, a general acceptance of TPT, was overly simplistic. The two groups, while contrasting in the overall outcome of completion or discontinuation, were more similar in their experiences navigating the challenges associated with TPT. Those who did complete their most recent course of TPT rarely described a journey free of challenges. This meant that comparing the broad experiences of the two groups was less analytically informative than we had anticipated. It was more productive to explore and examine TPT acceptability as informed by biosocial challenges that take place in a context of AYPLHIV’s shifting circumstances and priorities.

To represent this complexity, our findings are organised by the following themes: 1. Tensions between TPT explanations and experiences; 2. Fluid and heterogeneous assessments of TB risk based on evolving experience and knowledge; 3. TPT in the context of comorbidity. Sub-themes within each describe the nuanced experiences and perceptions of TPT, situated within participants’ changing individual circumstances and the broader social narratives surrounding HIV that shape relationships with treatment adherence.

### Tensions between TPT explanations and experiences

Participants described healthcare workers (HCWs) introducing TPT as a routine and uncomplicated intervention that runs parallel to, and does not interact with, HIV treatment. It was presented as a simple but important process for protecting themselves, their households, and their communities, which was interpreted by several participants as conveying their individual moral responsibility to take up the offer: *“I knew I ought to take it”* (22-year-old male, PID 06, FGD 2). In contrast, most participants experienced TPT as daunting*,* difficult*,* and oftentimes disruptive, commonly citing concerns related to their existing challenges with managing their HIV.

*“The information that I got from nurses did not impact my decision but the side effects I experienced did. It was a ‘no’ for me” (22-year-old male, PID 06, IDI).*

*“I’m on second line ART treatment. So having to take TPT was adding to this burden” (24-year-old female, PID 02, IDI).*

The impact of this tension altered participants’ perceptions of TPT, and potentially of those who had advised them to take it: *“they said it was something that was meant to help us”* (22-year-old male, PID 11,IDI).

#### Resurfacing old narratives: The moral weight of treatment compliance.

A notable reluctance among participants to speak openly about their challenges with TPT amongst their peers was also evident. This contrasted with this group’s usual candour when speaking about difficulties with ART adherence, which has been normalised in Zvandiri support groups. The way participants spoke about their experiences navigating TPT suggests that the process of normalising discussions around TPT challenges has yet to happen, which meant that participants did not find it as easy to share their difficulties or to seek support. This was particularly evident in the FGDs, where those who later revealed significant challenges in their IDI were reluctant to speak about their personal experiences in front of others. When it did become apparent in the FGDs that challenges with taking TPT were not uncommon, this had a reassuring effect. For example, in a later individual interview, one FGD participant described the feeling of discovering that she was not the only one who had discontinued treatment as *“the guiltiness in me disappearing”* (23-year-old female, PID 04, IDI).

Although TPT was presented to participants as a choice, several participants’ accounts reinforced this idea of moral irresponsibility if they had failed to complete TPT, especially when they believed they were the only one amongst their peers.

*“I just knew myself that it was wrong … So, I kept quiet about it. I also thought I would be the only person who was not taking it. I thought everyone else would be taking it. So, I just decided not to talk about it” (24-year-old female, PID 02, IDI).*

### Fluid and heterogeneous assessments of TB risk based on evolving experience and knowledge

Whether participants had a positive or negative attitude towards TPT was significantly influenced by i) their prior knowledge and experience of TB, and ii) how this interacted with their HIV-related concerns and priorities. For those with close personal experience of TB illness, such as witnessing a family member suffer or take treatment, this knowledge could override negative preconceptions and could even temper the experience of some of the milder TPT side effects.

*“One of my family members got TB and I got to witness the pain and the weight loss. The way they were coughing, and their jaws were shaky. That’s when I decided to start taking TPT” (23-year-old female, PID 07, FGD 2).*

It was common among those who had witnessed a family member experience TB illness and treatment to have a more favourable view of short-term engagement with TPT.

*“Rather than taking TB pills, they are too many. Instead, just take one pill per week and then it’s done” (23-year-old-female, PID 07, IDI).*

For this participant who had witnessed the burden of TB treatment, TPT was perceived as a much easier and more manageable prospect. However, those without direct experience of TB illness tended to view the combined pill burden of TPT and ART as overwhelming.

*“When I picked them up, I saw that there were many pills. At that time, I was told to take two and they were big, and my other pill to take at night, and also not forgetting my ‘Cotri’ [cotrimoxazole], aaah I decided to stop” (23-year-old female, PID 04, IDI).*

Participants without direct experience of TB were also more likely to be influenced by others’ accounts of side effects. The anticipation and fear of experiencing these symptoms often outweighed the benefits that had been presented to them by HCWs.

*“It was the side effects that I kept hearing from others. There were so many things that people were saying. I was scared, that’s when I decided to stop” (19-year-old male, PID 03, IDI).*

For several participants with direct experience, the initial motivation to prevent TB diminished over time. While they still felt strongly about wanting to avoid TB, other concerns such as how potential side effects might affect them socially, took on equal or greater significance, impacting their likelihood to take up subsequent courses of treatment. This was exemplified in the case of a 22-year-old participant (IDI06) who completed his first course of TPT in 2016 after witnessing his uncle struggle with TB. This very recent experience was a major motivating factor at that time, yet he declined two subsequent routine courses of TPT in 2019 and 2022. In 2016, this participant, who was administered a 6–9 month daily regimen, experienced side effects but managed to complete his treatment. In 2022, he was offered the shorter and more tolerable 3HP regimen; however, he was still deterred by his earlier experience. Here, he explains how he balanced the risks:

*“Yes, I was still scared of getting TB, but the issue of side effects made me decide to stop” (22-year-old-male, PID 06, IDI).*

#### Perceptions of risk to self and others.

There was an ongoing recalibration that appeared to shape participants’ continual adjusted assessments of the risks and benefits of TPT. This was not only calculated according to whether they had direct experience of TB illness, but also the recency of that direct experience. While for PID 06 the perceived benefit of TPT diminished, for others it increased. There were several participants who had described an initial reluctance to take TPT but revised their perceived need when a change in their work environment increased their contact with TB-affected patients.

*“There are many people who come to the clinic, including many TB patients who will be coughing and everything. I realised that I am at a heightened risk, so it would be better for me to start taking TPT” (23-year-old male, PID 08, IDI).*

For several participants, including PID 08, who started a course of TPT and did not experience serious side effects, this motivation continued to completion.

Participants’ shifting risk assessment was in part explained by their increased awareness of TB and TPT, which was integrated into their peer counsellor (CATS) training: “*… because I was now a CATS, I had more knowledge about TPT”* (19-year-old male, PID 03, IDI) and their alertness to their relational responsibilities, i.e., how their decisions could potentially impact the health of others they care about.

*“You will also fear for people at home. Because once I contract TB, I will be putting them at risk” (23-year-old-female, PID 07, IDI).*

Informed decision-making, and consequently a more positive sentiment towards TPT, was most evident when information describing the TB risk was tailored to participants’ personal circumstances. For example, one female participant who was pregnant at the time of TPT introduction felt ambivalent about TPT until she was provided information about TPT through Zvandiri’s Young Mentor Mothers Programme.

*“We had a training at Zvandiri where we discussed this topic. It also included information for pregnant mothers. So that is the other thing that pushed me to go and take them for the sake of my baby” (23-year-old female, PID 10, IDI).*

#### Proximate and distant risks: More than the risk of TB-illness to consider.

For participants who perceived the risk of TB as distant due to a lack of experience or sense of peripheral exposure, the immediate unwelcome impacts that TPT side effects could have on their physical appearance, peer relationships, and social wellbeing were the more compelling influences on their decision-making. This balancing of risks was clearly socially mediated, with numerous examples of the persuasive effect, both to take up or to discontinue TPT, of negative stories about TPT that were circulating within their social networks.

*“Yah, people were saying that they can make you sick, others saying they make you feel hungry, a lot was being said” (23-year-old female, PID10, IDI).*

*“Others alarmed me when they told me that they were pills that ward off evil spirits and that if I did not take them, I would get sick (chuckles), so I decided to start taking them” (23-year-old female, PID 09, IDI).*

*“Others were saying it makes you dark and all that. There were so many things that people were saying. I was scared of all that, that’s when I decided to stop” (19-year-old male PID 03, IDI).*

The lack of emphasis given to side effects by HCWs and the often-generalised explanation for the need to take TPT tended to jar with participants’ limited discussion about TPT with their peers, which concentrated almost exclusively on side effects. Some participants suggested that HCWs withheld information about side effects so as to not dissuade their uptake of TPT. Paradoxically, this seemed to undermine participants’ confidence in the ‘official’ healthcare advice about TPT. Coupled with a propensity to trust their peers, this endowed the narrative of difficult side effects with considerable credence. Such was the power of collectively shared stories, that one participant discontinued TPT and adopted her friend’s story about side effects, even though she had not experienced them personally.

*“I would share with my friends that I hung out with that these pills that I was given make me feel hungry and dizzy, but I have never felt dizzy” (23-year-old female, PID 05, IDI).*

TPT side effects carried social risks, which manifested as experienced and anticipated stigma. This was a major disincentive for TPT uptake. Visible side effects, such as skin darkening or yellowing of the eyes, were common reasons for forgoing or discontinuing TPT, whether personally experienced or not. Young women were particularly concerned that TPT might darken their skin, reflecting the social desirability for lighter skin in Zimbabwe.

*“You know people talk a lot in the community saying TB pills cause one to become darker in complexion, becoming a “black person”. So, when I first heard about it, I thought that I cannot become darker when I am dark already, so I decided to delay taking them” (23-year-old female, PID 07, IDI).*

### TPT in the context of comorbidity

It was evident in many participants’ accounts how closely their experiences and perceptions of TPT were entangled with their past and present experiences of managing HIV. For several participants, TPT side effects that echoed their earlier experiences of symptoms associated with unmanaged HIV triggered unpleasant memories of stigma and discrimination.

*“It drew me back because I last had fever and allergies (skin reactions) a long time ago (due to unmanaged HIV). Back then I had herpes, which caused people to discriminate me. So, I just started thinking, why did I take this (TPT) in the first place?” (22-year-old male, PID 11, IDI).*

This participant’s ART regimen had not changed, and the symptoms he described were side effects of TPT that reminded him of times when he had struggled to consistently take ART. For this participant, who discontinued treatment prior to completion, the purpose of TPT was outweighed by the very real short-term detrimental impacts on his health and wellbeing.

PID 06, who had previously completed TPT, described similar impacts due to the timing of his latest TPT course. TPT was reintroduced when his viral load was detectable, and his mental health was unstable. His attempt to take TPT at this time had negative consequences that informed his decision to discontinue.

*“Now that I took this TPT, it’s affecting my (ART) adherence, viral load, even my wellbeing in the community” (22-year-old-male, PID 06, IDI).*

#### Looking and feeling healthy: TPT side effects contradict established treatment adherence narratives.

A dominant concern among participants was how TPT side effects contradict what they have come to understand about the positive, amplifying effect of treatment engagement on their health and wellbeing, gained through their engagement with Zvandiri. One participant who decided to discontinue TPT described how it caused her to feel sick, which alerted her that something was wrong. Her positive experiences of achieving viral suppression from sustained ART informed her interpretation of the symptoms she was experiencing and its relationship with adherence.

*“As a person who is on ART, personally I don’t like being sick… So, whenever I feel any pain, I feel like there is something that I didn’t do right” (23-year-old-female, PID 09, IDI).*

TPT enters this dynamic and complex relationship between treatment adherence and embodied experiences of health and illness. Many TPT side effects, such as weakness and tiredness, nausea and vomiting, headaches and skin blemishes, echo physical symptoms of inconsistent ART and a high viral load. Experiencing these symptoms whilst adhering well to a treatment, as they were with TPT, is antithetical to how ‘good adherence’ should look and feel.

#### Experienced side effects are a risk to CATS’ sense of purpose and self-efficacy.

Participants also described how side effects affected their capacity to function effectively: *“I would feel weak to the point that I couldn’t do anything” (22-year-old male, PID 06, IDI)*, which impacted their ability to fulfil their responsibilities, including their peer-support role at Zvandiri. Participants also shared their concerns about the unintended influence that non-completion of TPT could have on those to whom they provided peer support. They described how integral authenticity and honesty, particularly around ART adherence, was in their relationships with young people. When it came to TPT, participants felt that they had to provide advice that contradicted their own experience. This represented an uncomfortable tension which risked corroding their self-efficacy, with several commenting on the moral predicament that this posed. Their desire to maintain their good role-model image led many to hide their decision to stop TPT from most people, including those within Zvandiri from whom they might have been able to seek additional support. This risked undermining the trustworthiness of the dialogue that had been so thoughtfully and successfully cultivated taking in peer support relationships.

*“I realised that what I was doing was wrong. I am a person who supports others in taking TPT, while I fail to do the actual right thing” (24-year-old female, PID 01, IDI).*

*“We have to lead by example because we are the ones who will be encouraging other adolescents to take TPT at the same time we are the ones that are refusing to take it” (23-year-old female, PID 04, IDI).*

## Discussion

This study has shown that participants’ experiences and perceptions of TPT are individual and circumstantial, and shaped by shifting concerns related to the continued management of HIV. The acceptability of TPT among participants was significantly socially mediated, with narratives about TPT having both a dissuading and persuading effect, depending upon individual concerns and priorities. HIV was often implicit in participants’ evaluation of TPT, whether it was in how they assessed risk and pill burden, or how they responded to stigma and side effects.

For several participants, TPT exacerbated mental health challenges, which then threatened to undermine their ART adherence. This echoes research that has established that taking TPT can contribute to a decline in mental health and wellbeing [34,35]. Coupled with what we know about the relationship between mental health and HIV outcomes [36,37], which is most apparent in unsteady adherence practices, it is clear that the cumulative burden of managing both requires the utmost attention and support. Given that participants were all trained peer-supporters with high health literacy and specific training in TPT, this finding carries considerable gravity. The (proximate) social risks of TPT side effects, whether experienced or anticipated, were often more influential in participants’ decision-making than the (potentially distant) risk of potentially developing TB-illness. The lack of recognition in the clinical presentation of TPT hampered their access to support in navigating these dilemmas and led many participants to forgo or discontinue TPT, often without sharing this decision.

As is common in public health preventive efforts, the presumption of ‘simplicity’ in how TPT was introduced was interpreted by participants as conveying their individual moral responsibility to take up the offer of this preventive opportunity. This is echoed in how young people have previously described the moral weight of not adhering to ART [38]. As is the case with the moralisation of ART adherence, participants in this study described feeling compelled to remain silent about their struggles to adhere for fear of being judged. For participants in this study, the perceived need for silence was compounded by their role as peer counsellors in role-modelling treatment adherence, which underpins their positive influence on beneficiaries.

The recent change to how TPT will be administered in Zimbabwe, which differs from the circumstances under which the study was conducted, is welcome. Limiting TPT to those with a known exposure, or a positive test for infection, will reduce much of the burden described by participants in this study. Parsimonious TPT protects the willingness of AYPLHIV to adhere and should be reserved for situations when TPT is clearly warranted. However, for this group, who work closely with households at higher risk of TB, and who themselves share overlapping vulnerabilities with the young people and their families whom they support, *M.Tuberculosis* (re)exposure is likely to remain consistently high [39]. Managing their risk is not only about curtailing progression to TB disease and protecting scarce and precious healthcare worker resources, but also about limiting the risk of onward transmission within the community. It is likely then that the public health logic in encouraging AYPLHIV to engage in TPT continues.

If TPT is predicated on known exposure to *M. Tuberculosis* and/or a positive test result for infection, young people will at least have a clearer understanding of the need for treatment. However, the issues associated with side effects, and stigma, remain a concern even if preventive treatment occurs less often. This study has highlighted several opportunities to improve and strengthen the communication and support that accompanies TPT delivery to AYPLHIV, that remain pertinent under the new guidelines.

TPT is a vitally important and promising clinical innovation that is being successfully integrated within HIV differentiated service delivery models in high-burden settings [40–42]. However, for it to be effective, especially in meeting the specific needs of AYPLHIV, it needs to be accompanied by appropriate tailored support that enables AYPLHIV to make informed decisions about their health. Failing to provide this support not only heightens the likelihood of TPT non-completion; it also risks ART adherence and increases the risk of both developing TB illness and poor HIV outcomes.

### Making a sustainable START to TPT

**Fig 1:**
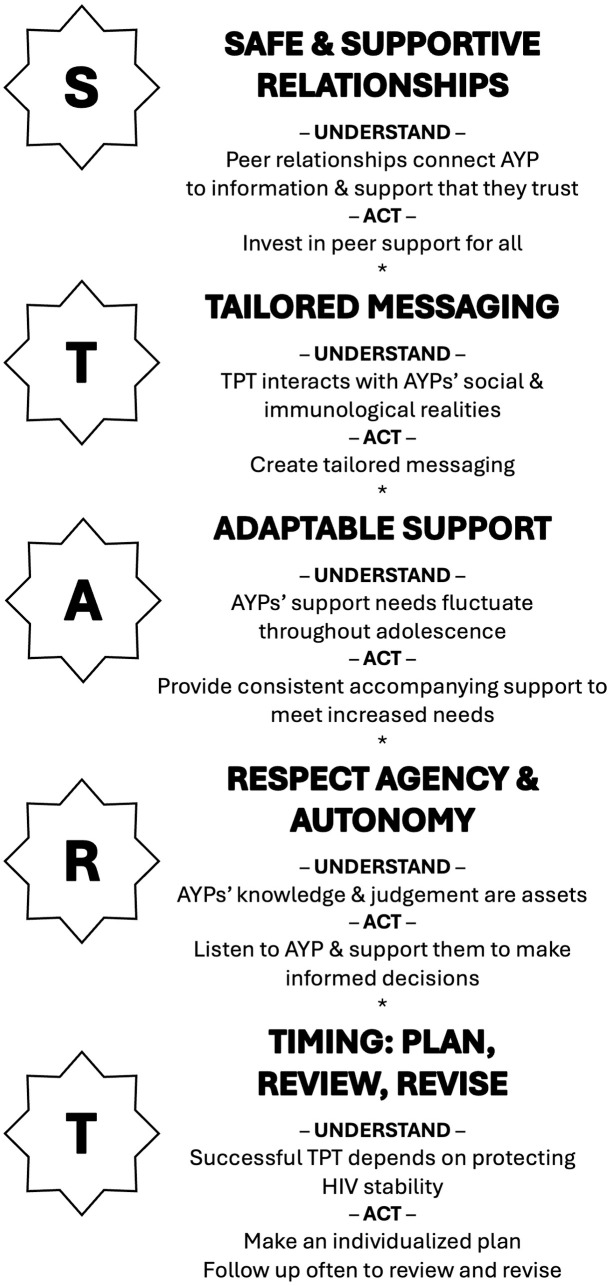
START Guiding principles for providing tailored integrated treatment support for adolescents who are living with HIV prior to and during TPT.

To support these efforts, we have developed a set of guiding principles, called START (Fig 1): Supported and safe relationships; Tailored messaging; Adaptable support; Respect for agency and autonomy; Timing: plan, review, revise. START is designed to guide the design and delivery of tailored and integrated treatment support for AYPLHIV prior to and during uptake of TPT. The principles convey the need for a person-centred and rights-based approach to the provision of care [33] and emphasise the value of establishing or utilising existing peer-peer relationships as a vital mechanism for supporting AYPLHIV to navigate TPT alongside ART, with opportunities to openly manage challenges as they arise.

This model was developed based on the findings from a very specific group and context. Its principles though are more widely relevant and could be adapted to support the introduction and integration of other supplementary health interventions for AYPLHIV in Zimbabwe and in other contexts, as well as for any age group, including adults.

#### Supportive and safe relationships.

Safe and supportive relationships are critical conduits for connecting AYPLHIV to the information and support they need and trust [43]. Such relationships empower AYPLHIV to voice concerns, ask questions, advocate for themselves, and make informed decisions about their health [43]. This study draws attention to the potential value of trusted peer support networks to support TPT uptake. Where these networks are yet to be established, non-peer support should be integrated within each stage of TPT to ensure that young people are not alone or isolated in their decision-making.

In this study, we engaged trained peer counsellors with high health literacy. Even so, they each struggled to ask for the support that they needed. This contrasts with how forthcoming they typically are when they encounter ART-related challenges. Our findings suggest that this is likely because the challenges associated with TPT have not yet been normalised as has been achieved with ART, due to the years of dedicated effort by Zvandiri’s staff and peer network. To overcome this reserve, pre-existing supportive relationships that are already grounded in trust and non-judgement are key to helping AYPLHIV feel safe to express concerns and ask questions. When participants in this study became aware of their shared challenges, they described feeling reassured and more inclined to speak with their peers without fear of judgement. Safety is maintained through trust and non-judgement, and it is vital that the support offered engages with the dilemmas or trade-offs that are embedded within informed decision-making about preventive treatments.

#### Tailored messaging.

This research has shown that public health messaging to AYPLHIV surrounding TPT woefully under-appreciates the complexity of the contexts into which it enters. That, in this study, trained peer counsellors struggled with their own uptake of TPT, highlights the need for adequate tailored information and ongoing support for peer counsellors to ensure they can provide effective support to others.

Public health messaging must be adapted to reflect young people’s immunological and social realities. This means integrating discussions of TB prevention within existing HIV management; transparently communicating the benefits and limitations of TPT alongside other preventive methods; explaining the differences between TPT side effects and unsuppressed HIV; and discussing strategies for seeking additional support to manage disruptions to ART adherence and general wellbeing. It may also include, if feasible, offering testing to confirm whether a person is infected to assist in making a truly informed choice.

Supporting AYPLHIV to be better informed of the benefits and limitations of TPT is a necessary next step in improving TPT literacy. Given the workforce shortages and HCWs’ competing priorities [10], it may not be feasible to expect HCWs to take on this additional task of guiding TPT literacy. As it aligns closely with the existing provision of peer support, TPT literacy following the START principles can be readily absorbed, through additional training, by the peer counsellors. This is a clear example of the value of investing in the extended role of peer counsellors to deliver appropriate and tailored information, which can complement and support HCWs already functioning at capacity.

#### Adaptable support.

This study has highlighted a need for consistent support that is responsive to periods of fluctuating need throughout adolescence. TPT was disruptive for most participants because it intervened into a scenario where health and wellbeing were already fragile. Without a support mechanism to recognise fluctuations in ART adherence and wellbeing and respond accordingly, such interventions threaten to undermine stability and sow distrust. Zvandiri’s peer support model is so effective because it nourishes supporting relationships that are adaptable and responsive, and that evolve throughout adolescence. With further investment, peer counsellors can provide critical support to HCWs by playing a key role in enhancing TPT literacy, counselling and support. This is more than support; it is accompaniment, and it can adapt to be responsive to external changes of all kinds, including the introduction of a preventive intervention.

#### Respect agency and autonomy.

Key to supporting young people to make informed decisions is learning to understand, value and respect the knowledge that they have about their own health and wellbeing. Embodied knowledge (i.e., the bodily sense of how to live in one’s own body) is increasingly recognised as a critical resource for people who are living with a chronic condition that can be better utilised in healthcare delivery [44–46]. In this study, several participants described how their TPT engagement was influenced by their embodied knowledge of what it means to live well with HIV. Prioritising this knowledge in discussions about the clinical and public health benefits of interventions like TPT invites a fuller picture of how to support those living with a chronic condition to manage their health. Learning about health and wellbeing is bidirectional: young people bring their knowledge and expertise to the conversation, and professionals must make space for that knowledge to interact with official public health messaging and advice.

This means that whilst emphasising the benefits of TPT, we also need to respect the agency and autonomy of young people, by trusting and listening when they express concern for their health, in particular their ability to maintain ART adherence alongside additional treatments. This can only be achieved through open dialogue where young people’s self-knowledge and expertise are as valued (if not more so) as clinical expertise and public health priorities.

#### Timing: Plan, review, revise.

This study has shown that working with AYPLHIV to establish when TPT is least likely to be disruptive and developing a plan that coordinates increased support is critical to its success. For several participants, TPT was introduced when they were already experiencing challenges with ART adherence. TPT then compounded these challenges, risking both the individual’s ability to complete TPT and maintain ART adherence. Listening to AYPLHIV to understand the situation into which additional treatments are introduced, and co-creating an individualised plan, empowers them to choose the conditions that are right for them, and to be equipped with the support they might need as they progress with treatment. Ensuring opportunities to reassess and revise the plan is critical, particularly when side effects make sustaining both TPT and ART challenging.

Although TPT is time-bound, the START rubric echoes lessons from well-established HIV treatment initiation practices, supporting individuals to achieve sustained optimal outcomes [26]. Those learnings emphasise the need for HCWs to feel confident that they are introducing TPT in a way that sustains positive outcomes for TB prevention whilst maintaining HIV management stability. This requires a transparent description of the benefits and realities of TPT to enable young people to make an informed decision as to whether they continue or pause TPT to preserve their ART adherence. Recognising this informed health decision as a ‘positive’ outcome for these individuals is vital for ensuring the open dialogue necessary for revisiting TPT upon future exposure or once ART adherence has stabilised.

#### What this research tells us about how we approach ‘acceptability’ studies.

This study has also highlighted broader concerns related to how acceptability studies, both qualitative and quantitative, often pose the question of an intervention’s appeal to recipients in narrow, constrained, and prescriptive terms. Acceptability is often couched as a discrete choice, where participants are only granted the opportunity to provide feedback that might minimally adjust the components of an intervention or how it is delivered. A person-centred approach that employs appropriate frameworks and methods listens first to what recipients want and need from both clinical and psychosocial interventions, even and especially when they surpass what is assumed to fall within the remit of the intervention [47–49].

As explored in this research, a narrow focus on TB, the unproblematic additive effect of further treatment in the context of ART, and the presumed moral good of taking up the offer of TPT have limited the scope of what seemed legitimate concerns and questions. This leads participants to stay silent about their needs and their choices. Our inquiry made space for more expansive insights into how TPT interacts with existing ideas about health and wellbeing. This pushes us to interrogate: *Are acceptability studies framing enquiries in ways which engage with an individual’s broader context and their layered (often competing) priorities which influence decision-making? If such an approach is intrinsic to person-centred care, how are we responding to this if it collides with the public health logic to promote maximum uptake of preventive interventions? Are we utilising the methods at our disposal to enable these tensions to be shared, heard and responded to?*

### Strengths and limitations

Participants’ openness to share their challenges with TPT was facilitated by their longstanding relationship with Zvandiri, a programme that emphasises youth empowerment, trusting and supporting young people to manage their own health, and the destigmatisation of both HIV and adolescence. In this context, difficult subjects and experienced challenges are more sayable than in other environments. While the sample in this study was small, the recruitment of participants with exceptional health literacy and who trust in the programme through which they were recruited is a major strength. This study has particular significance for young people who are not yet engaged in peer support; it has underscored the value of continuing to invest in, and expand the availability of, peer support for all young people who are living with HIV.

## Conclusion

This research has highlighted the challenges experienced by AYPLHIV when a biomedical prevention intervention, such as TPT, is uncomplicatedly introduced alongside ART. As the World Health Organisation (WHO) call for the scale-up of TPT to meet their 2035 End TB targets, there needs to be accompanying investment in the supportive structures that enable TPT to complement rather than risk degrading the physical and mental health of AYPLHIV. TPT is a powerful tool that can meaningfully reduce the risk of developing TB amongst young people living with HIV [50]. Pill burden, inadequate information and side-effects make TPT a treatment that must be coupled with tailored treatment support in an ‘information healthy’ environment that respects patient autonomy. It behoves health systems, within their TB and HIV programmes, to create environments that give young people their best chance of protecting themselves and their loved ones from TB. This best chance STARTs here.

## Supporting information

S1 Text FGD topic guide with peer supporters who decided to take up TPT. (TIF)

S2 Text FGD topic guide with peer supporters who have decided not to take up or have not completed TPT. (TIF)

S3 Text IDI topic guide. (TIF)

S1 Checklist Inclusivity in global research questionnaire TPT. (DOCX)
