# Supplementary figures and images for: “Now that I took TPT, it’s affecting my ART adherence, viral load, even my wellbeing in the community”. Exploring acceptability and experience of Tuberculosis Preventive Treatment among adolescents living with HIV in Zimbabwe
Source: PLOS Glob Public Health. 2025 Nov 4;5(11):e0005102. doi: 10.1371/journal.pgph.0005102 (PMC12585034; doi:10.1371/journal.pgph.0005102)

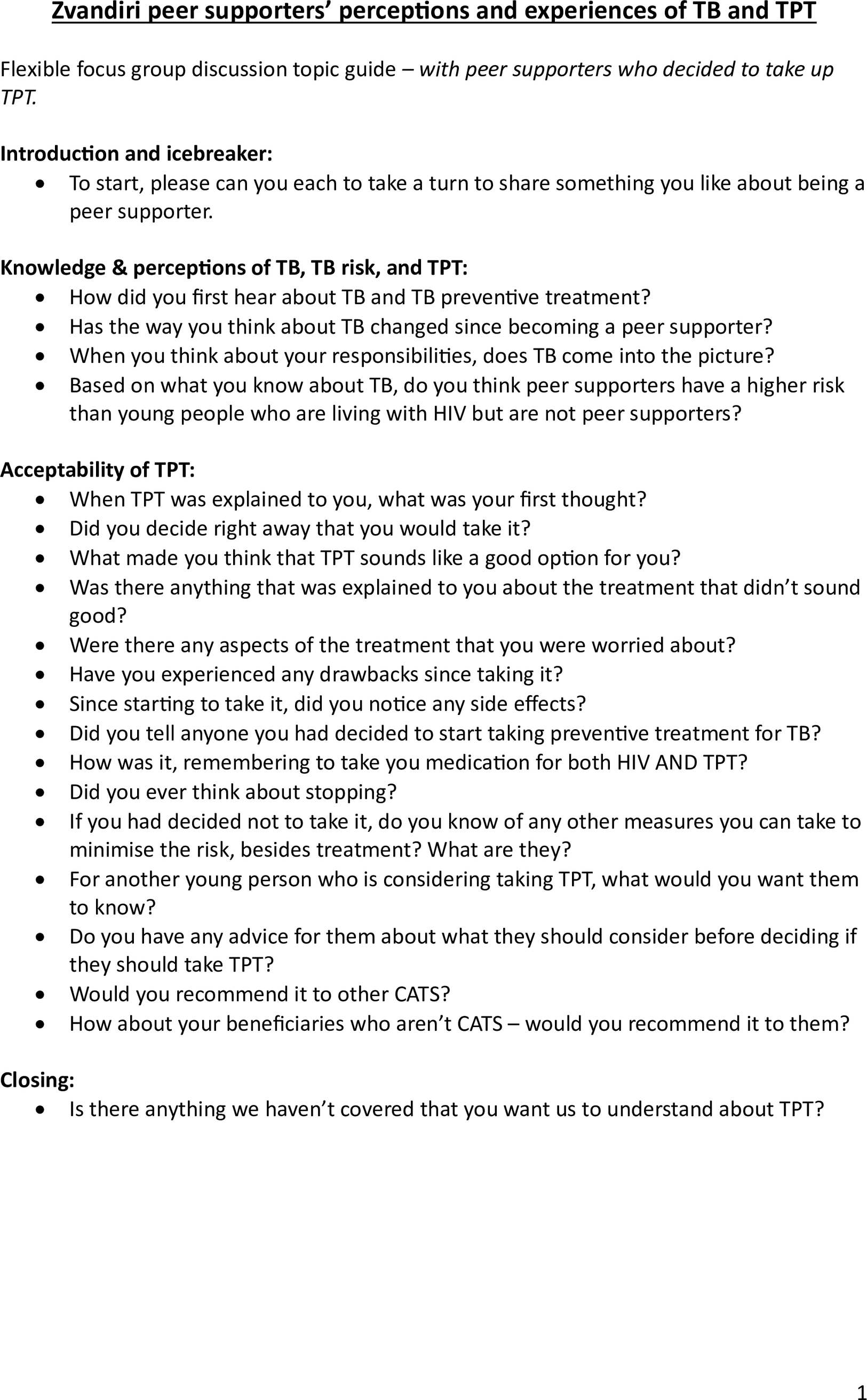

Supplement: S1 Text — (TIF) [file pgph.0005102.s001.tif]

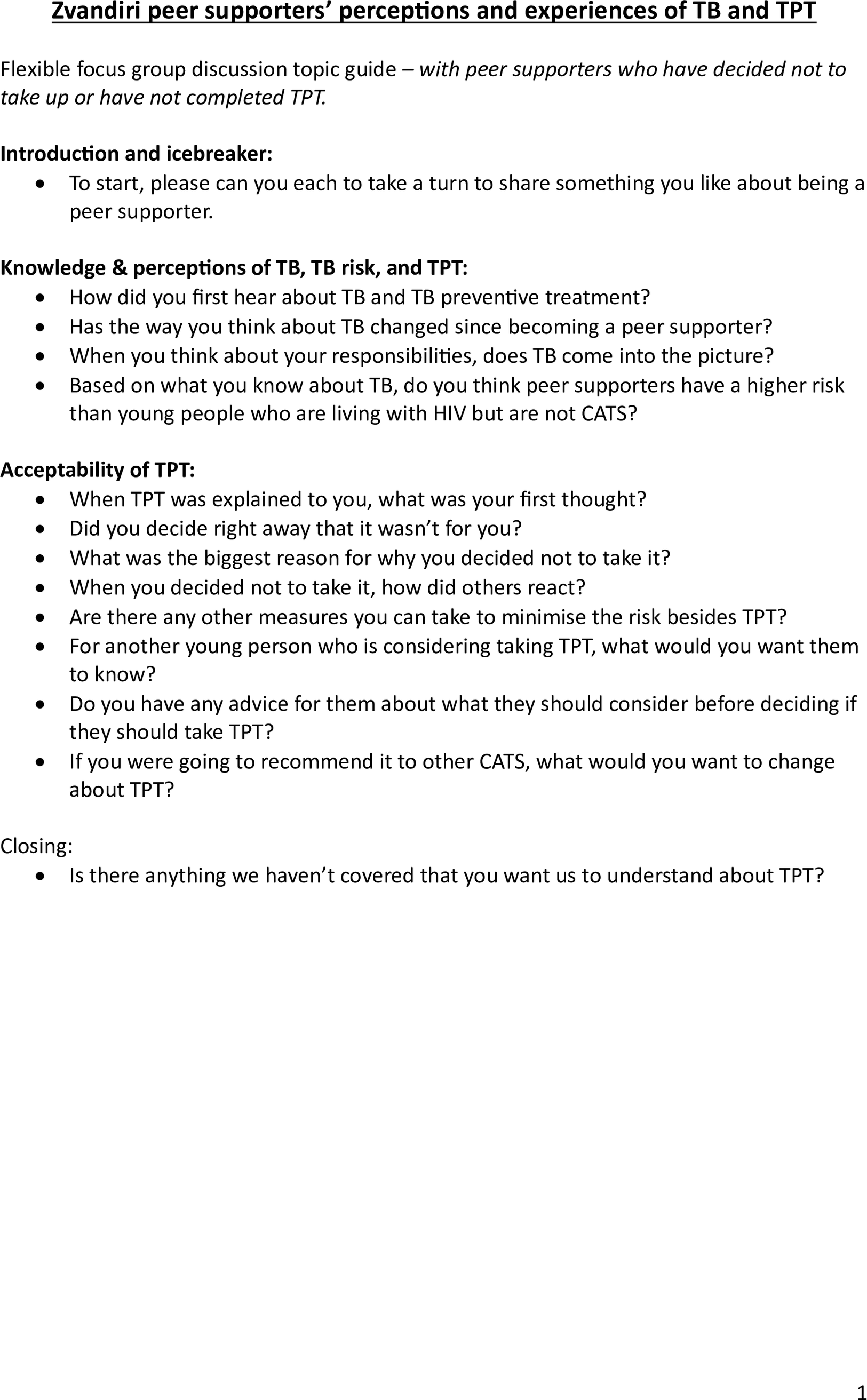

Supplement: S2 Text — (TIF) [file pgph.0005102.s002.tif]

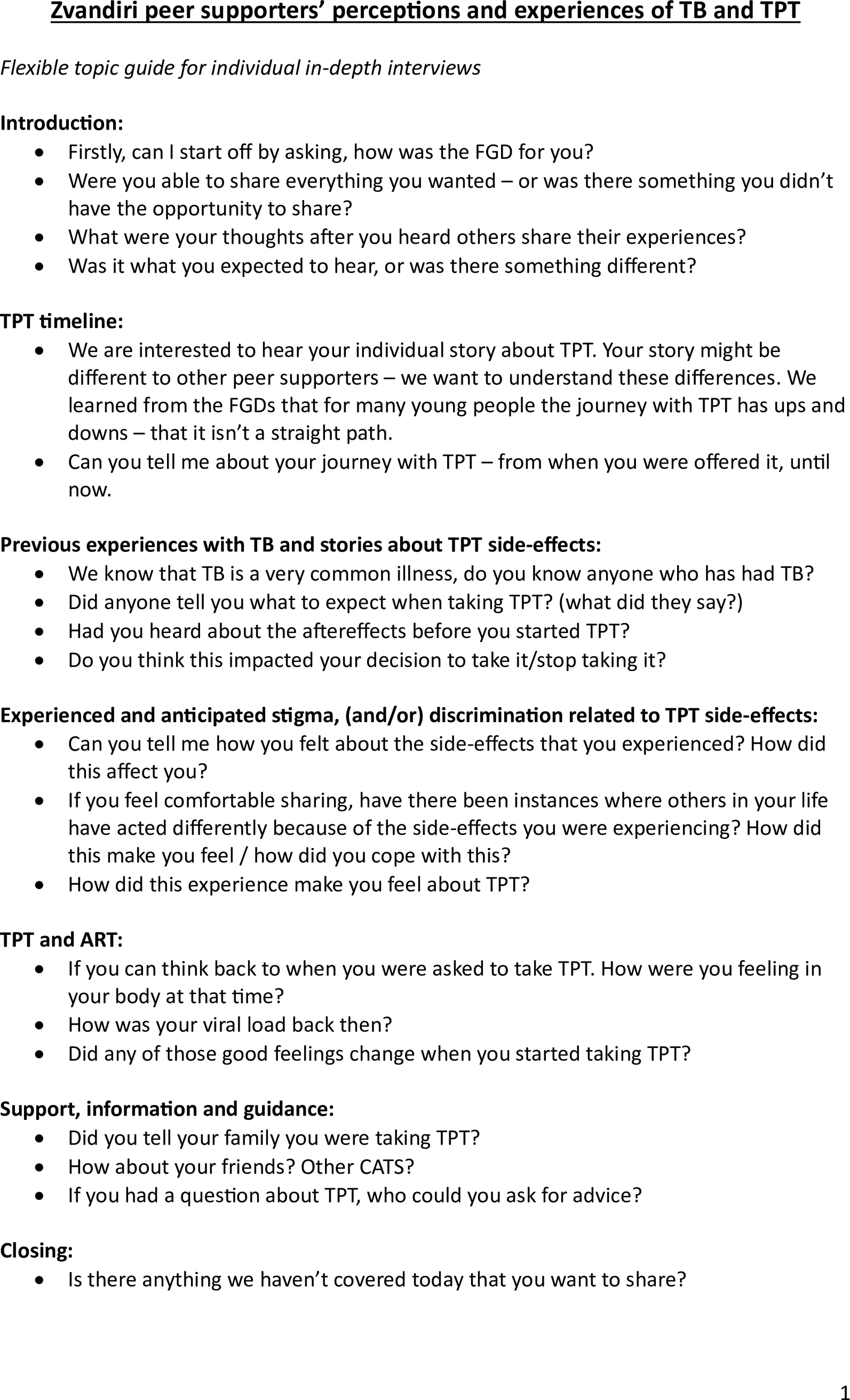

Supplement: S3 Text — (TIF) [file pgph.0005102.s003.tif]
